# Investigation of the Microstructural and Corrosion Properties of Steels and Light Alloys

**DOI:** 10.3390/ma16186171

**Published:** 2023-09-12

**Authors:** Luca Pezzato, Claudio Gennari

**Affiliations:** Department of Industrial Engineering, University of Padova, Via Marzolo 9, 35131 Padova, Italy; luca.pezzato@unipd.it

Very few metals can be found in metallic form in nature; the vast majority have to be processed from their ores at a great cost in terms of energy and money. It is therefore energetically favorable for them to reverse to their initial state. This process is commonly known as corrosion or anti-metallurgy, and great efforts are made worldwide to limit this process.

According to the latest NACE estimation (2013), the global cost of corrosion is equivalent to approximately 3.4% of the global GDP (2.5 trillion USD) not considering environmental consequences or safety issues. A reduction between 15% and 35% could be realized if prevention techniques and proper precautions are used, leading to savings between USD 375 and 875 billion. Corrosion involves different sectors, such as industry, military, civilian, services, etc., particularly energy production, transport, chemical and petrochemical industries, the mechanical industry, and the drink and beverage industry. Among these sectors, most of the constituents are made of steel, which is the most-produced metal in the world (1808 million tons in 2018) or light alloys, mainly aluminum (60.1 million tons of consumption in 2018).

A proper alloy design in terms of composition, heat treatment, microstructural features, etc., is mandatory to obtain the best combination of mechanical properties and corrosion resistance during operation, reducing maintenance costs and the overall impact on the global economy. In fact, microstructural features can affect both the corrosion of the material itself and the eventual production of protective layers on their surfaces.

The purpose of this Special Issue is to correlate the key role of the microstructure of steels and light alloys with their corrosion properties.

Maslak et al. [1] studied the impact fracture surfaces as indicators of structural post-fire susceptibility to brittle cracking in different steel grades (i.e., S355J2+N structural steel, X20Cr13 martensitic stainless steel, X6CrNiTi18-10 austenitic stainless steel and X2CrNiMoN22-5-3 duplex stainless steel). They found that various steel grades behave differently after surviving a fire incident, in a manner that is not obvious and difficult to foresee if not supported by appropriate specialized tests. They confirmed the need for a detailed analysis of impact strength to reliably draw conclusions regarding the possible future safe application of material that survived a fire.

Lim et al. [2] investigated a new Al-Mn-Zr alloy by means of microstructure and corrosion behavior after laser welding to a conventional AA3003. Thew found that laser welding caused interdendritic segregation and dissemination of intermetallic compounds throughout the fusion zone, this increased micro-galvanic corrosion sites, destabilizing the passive film. The addition of Zr reduced the size and number of intermetallic compounds minimizing micro-galvanic corrosion and enhancing the corrosion resistance compared to the AA3003.

Pezzato et al. [3] studied the effect of the addition of copper on the corrosion and antifouling properties of PEO-coated zinc-aluminized steel. They were able to successfully embed copper particles inside the PEO coating. The presence of copper particles enhanced the antifouling properties of the sample’s surface during the first 20 days of immersion in sea water. However, the corrosion resistance was negatively affected by the higher electrical conductivity of the PEO coating due to the presence of conductive copper particles, even though it remained higher compared to the untreated sample.

Khademzadeh et al. [4] developed a micro-laser powder bed fusion for the additive manufacturing of Inconel 718 alloy. They optimized the process parameters of the additive manufacturing machine to obtain the best microstructural properties for post-printing heat-treated Inconel 718. The result showed that using the optimum input energy density led to the homogenous distribution of nanosized (<10 nm) circular γ′ and plate-like γ″ particles in the γ matrix. When uniaxial tensile tests were conducted on heat-treated samples, they showed that ageing temperature is the most determinant factor in the mechanical strength of additively manufactured Inconel 718.

Yeh et al. [5] studied the stress corrosion cracking and crevice corrosion of AISI 304L stainless steel in a saline environment after the deposition of dust on the surface. They used a temperature of 45 °C and different relative humidities (45%, 55% and 70%) for 7000 h. They found that there is a relative humidity threshold (i.e., between 55% and 70%), above which SCC and crevice corrosion appear to have the lowest dust concentration. At the highest dust concentration, the relative humidity threshold is lowered between 45% and 55%.

Liang and Asselin [6] focused their work on the effect of bitumen at temperatures between 60 °C and 120 °C for 30 days on the corrosion of pipeline steel API X100. They analyzed inclusions by means of SEM EDS analysis and observed the effect of the exposure of the steel to bitumen on the matrix and inclusions of the steel. They found that bitumen under these conditions does not affect either the matrix or the inclusion of the steel.

Zong and Liu [7] worked on the microstructure, mechanical properties, and corrosion behavior of ultra-low-carbon bainitic steel with different niobium contents. They performed a tensile test, low-temperature impact toughness, corrosion weight loss method, polarization curves, and electrochemical impedance spectroscopy on four bainitic steels with different niobium contents. They observed that niobium affects the bainite morphology and the size, quantity and distribution of M/A elements. The impact toughness of all the samples at −40 °C is lower than 10 J. Better overall properties were observed in the steel with 0.0692% Nb.

Franceschi et al. [8] studied the effect of different austempering heat treatments on the corrosion properties of high-silicon austempering steel. They performed two sets of heat treatments: one changing the austenitizing temperature and keeping the austempering temperature constant, and the other keeping the austenitizing temperature constant and changing the austempering temperature. They also investigated the corrosion behavior of the treated steel in aqueous borate buffer solution to correlate the corrosion behavior with the microstructure arising from the various heat treatments that were performed. They were able to observe the highest volume fraction of retained austenite when the austenitizing temperature was 850 °C; moreover, the corrosion resistance increased when increasing the amount of retained austenite.

Yeh, Tsai and Huang [9] investigated the influence of chloride concentration on the stress corrosion cracking and crevice corrosion of austenitic stainless steel in saline environments. They analyzed different relative humidity value at 45 °C for 400 h and 10,000 h on the corrosion behavior of AISI 304L after spraying synthetic sea water on its surface. No crack was found on the speciman at 1 g/m^2^ of chlorine concentration after 400 h at any relative humidity value; instead, SCC cracks were observed at every relative humidity value for the specimen tested for 10,000 h. The chlorine concentration threshold required to initiate SCC in AISI 304L austenitic stainless steel at 45 °C and 10,000 h is between 0.1 g/m^2^ and 1 g/m^2^.

Biserova-Tahchieva et al. [10] conducted a comprehensive review of the additive manufacturing processes in selected corrosion-resistant materials. They critically discussed the corrosion resistance of light metallic systems and duplex stainless-steel objects obtained by additive manufacturing, highlighting methodologies that could improve this. Thanks to the data they collected, they concluded that the potentiodynamic corrosion tests are the best methodology to characterize metal additive manufacturing objects.

As can be seen from the papers published in this Special Issue, the collaboration of different institutions from all over the world was made possible, which helps to share the knowledge on, and increase the comprehension regarding, every subject.

As a concluding remark, the Editors want to thank all the authors and the editorial team of *Materials* for their collaboration in the peer review process. We genuinely hope that this Special Issue could improve the understanding of the link between the microstructure and corrosion properties of steels and light alloys.
